# Multiple roles and regulatory mechanisms of the transcription factor HNF4 in the intestine

**DOI:** 10.3389/fendo.2023.1232569

**Published:** 2023-08-10

**Authors:** Kiranmayi Vemuri, Sarah H. Radi, Frances M. Sladek, Michael P. Verzi

**Affiliations:** ^1^ Department of Genetics, Human Genetics Institute of New Jersey, Rutgers, The State University of New Jersey, Piscataway, NJ, United States; ^2^ Cancer Institute of New Jersey, Rutgers, The State University of New Jersey, New Brunswick, NJ, United States; ^3^ Department of Molecular, Cell and Systems Biology, University of California, Riverside, Riverside, CA, United States; ^4^ Department of Biochemistry, University of California, Riverside, Riverside, CA, United States

**Keywords:** HNF4, transcription factor, intestine, redundancy, intestinal differentiation, intestinal regeneration, colon cancer, inflammatory bowel disease

## Abstract

Hepatocyte nuclear factor 4-alpha (HNF4α) drives a complex array of transcriptional programs across multiple organs. Beyond its previously documented function in the liver, HNF4α has crucial roles in the kidney, intestine, and pancreas. In the intestine, a multitude of functions have been attributed to HNF4 and its accessory transcription factors, including but not limited to, intestinal maturation, differentiation, regeneration, and stem cell renewal. Functional redundancy between HNF4α and its intestine-restricted paralog HNF4γ, and co-regulation with other transcription factors drive these functions. Dysregulated expression of HNF4 results in a wide range of disease manifestations, including the development of a chronic inflammatory state in the intestine. In this review, we focus on the multiple molecular mechanisms of HNF4 in the intestine and explore translational opportunities. We aim to introduce new perspectives in understanding intestinal genetics and the complexity of gastrointestinal disorders through the lens of HNF4 transcription factors.

## Introduction

The specification of cell types and patterning of tissues depends on transcription factors which interpret and effectuate regulatory sequences of DNA. In this review, we explore the functions of a key transcription factor family in the intestine, HNF4. The intestinal epithelium is one of the most rapidly self-renewing tissues in mammals. The inner surface of the intestine is lined with a single cell layer of tightly packed, columnar epithelial cells. The epithelium is further organized into finger-like protrusions called villi which increase the surface area of absorption, and invaginations into the submucosa known as the crypts of Lieberkühn (1). The large intestine or colon has crypts but no villi (2). The crypts in both the small intestine and colon are characterized by a population of self-renewing, undifferentiated cells, which give rise to differentiated cell types: goblet cells, enteroendocrine cells, Paneth cells, tuft cells, and enterocytes (1). Goblet cells and enteroendocrine cells secrete mucus and a variety of hormones, respectively, and reside in both villi and crypts. Tuft cells are chemosensory cells which have been postulated to mediate defense against parasitic infection by releasing IL-25 (3, 4). The most abundant cell type in the villi, however, are the enterocytes, which are responsible for nutrient absorption. Each enterocyte has a tightly organized array of 1–2μm microvilli projecting from the apical cell surface that collectively forms the brush border and dramatically increases the surface area of this absorptive epithelium (5).

Meanwhile, in the crypts, Paneth cells occupy the base of the crypts and secrete antimicrobial agents such as α-defensins and lysozyme (6). They also participate in epithelial maintenance by expressing some of the canonical ligands of the Wnt and Notch signaling pathways (7). Additionally, the base of the crypts has a population of undifferentiated, multipotent stem cells marked by *Lgr5*. These crypt-base-columnar stem cells either renew and remain at the crypt bottom or differentiate into secretory cells or enterocytes which then migrate upward from the crypts towards the tips of the villi (8). Cells at the villus tips ultimately undergo anoikis as they are shed into the intestinal lumen (9, 10). Homeostasis of the epithelium is tightly regulated at the transcriptional level. It has been estimated using RNA-seq that nearly 4,000 genes are differentially expressed between the duodenal crypt and villus compartments (11). Several transcription factor families such as TCF/LEF, KLF, CDX (12–14), GATA (15), and HNF4 (16, 17) have been identified as important regulators involved in the control of these differentially expressed genes. In particular, HNF4 plays pivotal roles in regulating intestinal maturation, development, differentiation and architecture. We aim to provide an overview of HNF4 transcription factors, with specific focus on its role in the intestine.

## HNF4-mediated regulation in the gastrointestinal mucosa

**Figure 1:**
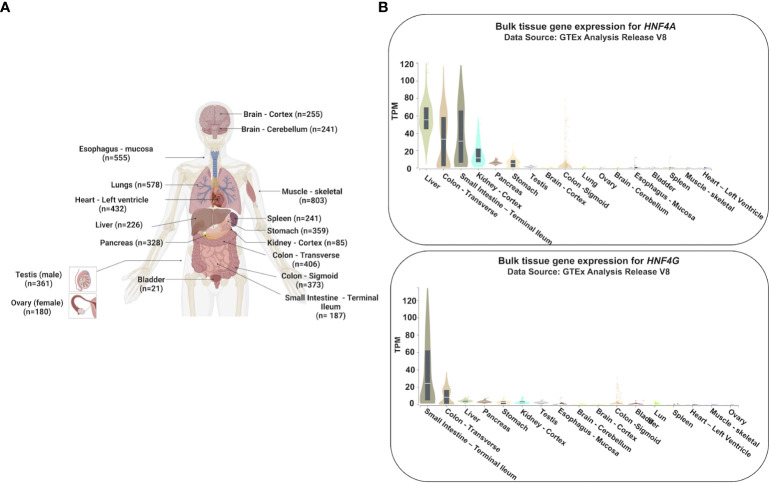
Tissue-specific gene expression profiles of *HNF4A* and *HNF4G.* **(A)** Expression profiles of *HNF4A* and *HNF4G* from the Genotype-Tissue Expression Project (GTEx) were evaluated in tissues of healthy individuals (n=number of replicates per tissue examined for gene expression) (20). **(B)** *HNF4A* is expressed across multiple tissues of the gastrointestinal tract whereas *HNF4G* is primarily an intestine-restricted paralog (TPM: Transcripts Per Million). The Genotype-Tissue Expression (GTEx) Project was supported by the Common Fund of the Office of the Director of the National Institutes of Health, and by NCI, NHGRI, NHLBI, NIDA, NIMH, and NINDS. The data used for the analyses described in this manuscript were obtained from the GTEx Portal on 05/25/23. Adapted from “Human Internal Organs”, by BioRender.com (2023).

Hepatocyte Nuclear Factors (HNFs) were identified based upon their abundance in liver extracts and ability to bind regulatory elements of liver specific genes (18). However, HNF4 is not just liver specific but is broadly expressed throughout the gastrointestinal system (19, 20) (**Figure 1**). The HNF4α homodimer binds via its DNA binding domain to its canonical DNA recognition site, DR1 (direct repeat 1; AGGTCAxAGGTCA) (21) and recruits co-regulatory proteins which mediate the regulation of its target genes. The first two identified targets of HNF4 activity were *Ttr* (transerythrin) and *Apoc3* (apolipoprotein C3), found using crude nuclear extracts of rat liver (18). Since then, HNF4 has been shown to perform crucial tasks in the liver, intestine, pancreas and kidney during development, differentiation, cell proliferation and for maintaining homeostasis. Tissue-restricted expression is highly conserved among species (22). The existence of two redundant paralogs of HNF4 (HNF4α and HNF4γ), along with their ability to isomerize and form different combinations of homodimers and heterodimers, adds significant complexity to the regulation of HNF4 and its target genes (23–25).

### HNF4 activates enhancer chromatin and facilitates long-range chromatin interactions at its target genes

Transcription of genes typically requires multiple events: i) the generation of active and accessible chromatin at one or more enhancers, ii) looping of enhancers to transcriptional start sites (promoters), and iii) the recruitment and activation of the RNA polymerase holoenzyme at promoters to generate mRNA transcripts. HNF4 appears to be involved in multiple steps of transcriptional activation of its target genes. An investigation of the HNF4α protein interactome with a BioID quantitative mass spectrometry experiment showed components of the ATP-dependent chromatin remodeling complexes SWI/SNF and NuRD, to be prominent interacting partners (23). This hints at HNF4α potentially mediating a permissive chromatin landscape. In fact, in the adult intestine this was shown to be the case as HNF4 factors maintain accessible, active enhancer chromatin (17). H3K4me3-targeted HiChIP in cells isolated from duodenal crypts and villi show HNF4 regulates its target genes by facilitating enhancer-promoter chromatin looping (11). A significant decrease in chromatin looping was observed upon loss of HNF4 factors, with far more HNF4-dependent chromatin looping events observed in the villus compared to the crypts. A multi-omics analysis incorporating H3K27ac micrococcal nuclease ChIP-seq inferred that HNF4 target genes depend upon HNF4 for chromatin accessibility at distal enhancer elements and for chromatin looping between enhancers and promoters. Functional annotation of these gene sets showed genes with disrupted chromatin loops upon HNF4 depletion were associated with steroid and lipid metabolic processes whereas genes with increased looping events were associated with a stress response. However, while HNF4 is required for local chromatin interactions, this is not a global cellular event, as genes without HNF4 binding did not show an appreciable change in chromatin structure on average (11). Further downstream of enhancer chromatin activation and chromatin looping, a potentially interesting avenue of investigation is the role of HNF4 in the recruitment and regulation of RNA polymerase II. In the intestine, little is known about RNA polymerase dynamics during gene transcription, but regulation of polymerase dynamics has the potential to be a new mechanism through which HNF4-dependent differentiation can be accomplished.

### HNF4 paralogs and their splice variants generate diverse expression patterns

The combination of HNF4 paralogs and their splice variants contributes to the complexity and versatility of gene regulation, allowing cells and tissues to fine-tune their gene expression profiles to meet specific requirements during development. The two mammalian paralogs of HNF4 – *Hnf4a* and *Hnf4g* are each expressed with distinct patterns across the GI system, and the gene products are further diversified through differential promoter usage and alternative splicing.

**Figure 2:**
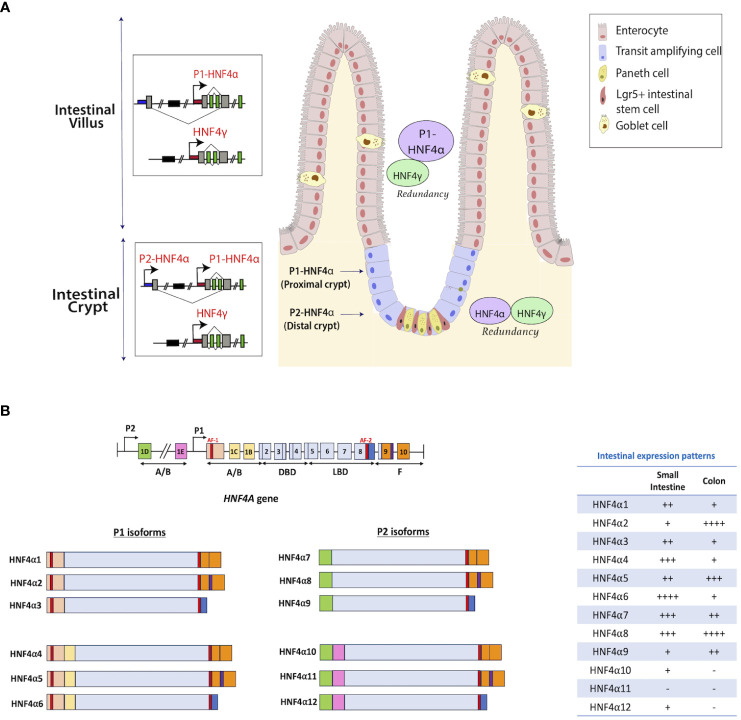
Distribution of HNF4 isoforms along the intestinal epithelium. **(A)** Isoforms of *Hnf4a* are generated through alternative splicing and usage of alternative promoters (proximal P1 and distal P2) that are forty kilobases (kb) apart in human and mouse (26). Enterocytes in the villi express high levels of P1 isoforms, whereas crypt stem cells express lower amounts (27). Studies in mouse and human colonic crypts show P1-driven HNF4α isoforms are expressed more robustly in the luminal, differentiated colonic epithelium, while P2-driven isoforms are enriched deeper in the proliferative crypt epithelium, co-localizing with the proliferative marker, Ki67. HNF4γ is expressed in both villus and crypt. **(B)** There are 12 isoforms of the HNF4A gene that are produced through the utilization of alternative promoters – P1 and P2. HNF4α1-6 are generated from the P1 promoter, while HNF4α7-12 are generated from the P2 promoter. The table shows the mRNA expression patterns of each isoform in the intestine from adult human tissue (25). Figure panel modeled off of the work of (25).

*Hnf4a* encodes multiple isoforms that exhibit variations in their N-termini. These isoforms are generated through alternative splicing events occurring at the 3’ end of the gene, coupled with the utilization of two distinct promoters, P1 (proximal) and P2 (distal), which are located approximately 40 kilobases (kb) apart in both human and mouse genomes (**Figure 2B**) (26). Notably, the epididymis and the intestine are the only adult tissues which express both P1- and P2- derived HNF4α in humans (28, 29). Studies in mouse and human colonic crypts have demonstrated that P1-driven *Hnf4a* isoforms are expressed more robustly in the luminal, differentiated colonic epithelium, while P2-driven isoforms are enriched deeper in the proliferative crypt epithelium, co-localizing with the proliferative marker, Ki67 (27, 29) (**Figure 2A**). Expression analyses of transgenic mouse models expressing either P1 or P2 isoforms (29, 30) demonstrate P1-HNF4α chiefly controls genes involved in differentiation, wound healing, and immune responses, whereas P2-HNF4α controls DNA repair and cell cycle genes (29).

Isoforms from promoter 1 (P1) include HNF4α1- HNF4α6 and are predominantly seen in the liver and small intestine with some expression also seen in the testis and kidney. Promoter 2 (P2) driven isoforms include HNF4α7- HNF4α12 and are seen in the pancreas, bile duct, stomach and GI tract (25). In both the small and large intestine, each isoform has distinct patterns of expression (**Figure 2B**). In the small intestine, HNF4α4, 6, 7, and 8 are expressed at higher levels, while HNF4α11 is not detected (25). Conversely, in the colon, HNF4α2, 5, and 8 show elevated expression, whereas HNF4α10-12 isoforms are absent (**Figure 2B**) (25). Differences were also identified in the transcriptional activation capabilities of the 12 isoforms. In HCT116 cells, upon individual expression of each HNF4α isoform using a tetracycline-inducible system, HNF4α1 and HNF4α2 demonstrated the highest effectiveness in regulating gene expression. Conversely, the transcriptional potential of HNF4α4, HNF4α5, and HNF4α6 isoforms was found to be diminished (23). Electrophoretic mobility shift assays showed the likely reason for the reduced transcriptional activity of HNF4α4, α5, and α6 is their inability to bind to the HNF4 consensus DNA binding sequence, DR1. The same study also showed that P2- isoforms exhibit a lower transactivation capacity than HNF4α1 and α2 (23). Interactions among isoforms can generate a repertoire of dimer combinations which influence numerous cellular processes.

In mammals, HNF4γ also exhibits two splice variants, HNF4γ1 and HNF4γ2. It is unclear if these are the products of differential promoter usage or alternative splicing. Expression of *Hnf4g1* is enriched in the kidney, intestine, and pancreas, whereas *Hnf4g2* is mostly intestine-restricted with the highest expression seen in the small intestine (31).

### Functional differences between HNF4α and HNF4γ proteins can be attributed to structural differences

**Figure 3:**
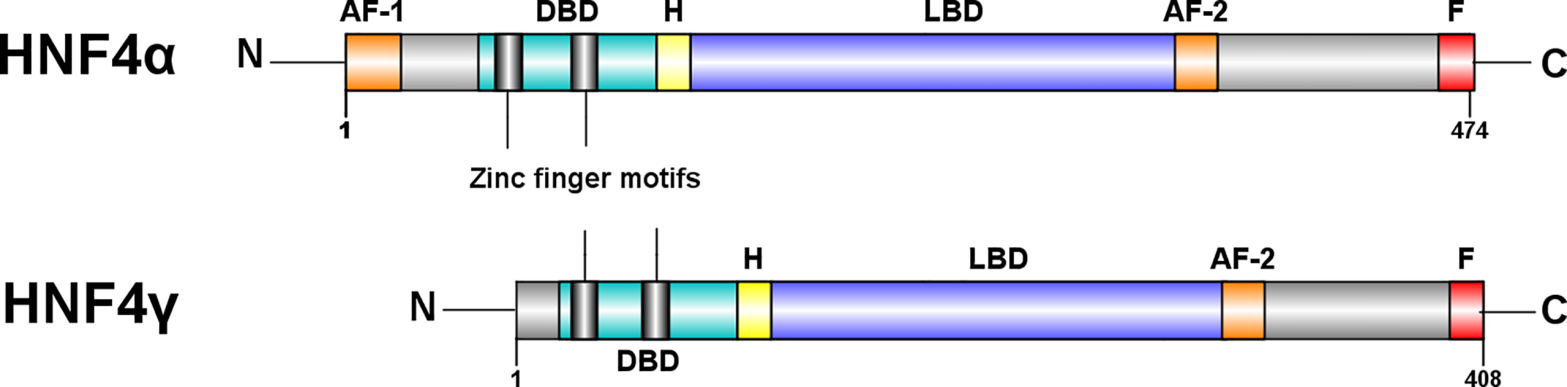
Structural differences between HNF4α and HNF4γ. HNF4α is typically a 474 amino acid protein amino acid protein, whereas HNF4γ1 is a 408 amino acid protein. Both paralogs have a DNA binding domain (DBD), a ligand binding domain (LBD), an AF-2 transactivation domain and a proline rich repressor domain (F) at the C-terminal. There is a 94% similarity between the DBDs and an 80% similarity between the LBDs (31). However, HNF4γ is missing an N-terminal, AF-1 transactivator domain. Figure generated using Illustrator for Biological Sequences, Version 1.0 (33).

HNFα and HNF4γ bind to many of the same sites in ChIP-seq studies of mouse intestine (17), and the two paralogs also have the ability to control specific target genes (17, 31). In humans, HNF4α ranges from a 392 to 474 amino acid protein depending on the isoform, whereas HNF4γ1 is made up of 408 amino acids (32) (**Figure 3**). In mice, the two paralogs, HNF4α and HNF4γ, share common features such as the ~200 amino acid ligand binding domain (LBD) and the 76 amino acid DNA binding domain (DBD), which are typical of nuclear receptors. However, HNF4γ1 lacks an N-terminal transactivation domain found in HNF4α and other HNF4γ isoforms. The C-terminal transactivation domain involved in coactivator binding, the AF-2 domain, is 100% conserved between the paralogs. The remainder of HNF4γ1 is highly similar to HNF4α, with a 94% similarity between the DBDs and an 80% similarity between the LBDs (34). In contrast, murine HNF4γ2 is a 448 amino acid protein with a functional AF-1 domain and no repressor domain (F domain). In many instances, the activity of the AF-2 region is suppressed by the repressor F domain (35). Hence, the lack of the F domain with a functioning AF-1 domain suggests that HNF4γ2 is a stronger transactivator than HNF4γ1 (31).

The lack of a specific ligand makes HNF4 an unorthodox nuclear receptor. Crystallographic structures of bacterially expressed HNF4α and HNF4γ showed their LBDs constitutively bind endogenous fatty acids (36, 37). In the bacterial-expressed HNF4 system, the bound fatty acids do not readily exchange other fatty acids or ligands and cannot be displaced without denaturing the protein (37). This suggests the fatty acid is trapped in the binding pocket during protein folding and cannot be separated from it. After translation, HNF4 spontaneously adopts a transcriptionally active conformation upon binding of an endogenous fatty acid in its ligand binding cleft. The fatty acid is presumed to act as a structural co-factor for HNF4 by facilitating the formation of a hydrophobic cleft in the LBD and stabilizes the α helical conformation of the protein, which allows it to bind to DNA and regulate its target genes (37, 38). However, in mammalian cells, HNF4α’s LBD was found to be reversibly occupied by linoleic acid (38, 39). There have also been reports of HNF4 binding to fatty acyl-coA thioesters, but their effect on HNF4’s regulatory mechanisms are uncertain (40). Expression profiling in HCT116 colon cancer cells revealed ligand occupancy does not impact the transactivation potential of HNF4α; in fact the presence of a ligand modestly represses HNF4α activity (39). Hence it is unlikely that HNF4 is dynamically regulated by ligands, but rather by post-translational modifications and interactions with co-regulators.

### Post-transcriptional modifications of HNF4 alter its regulatory landscape

**Figure 4:**
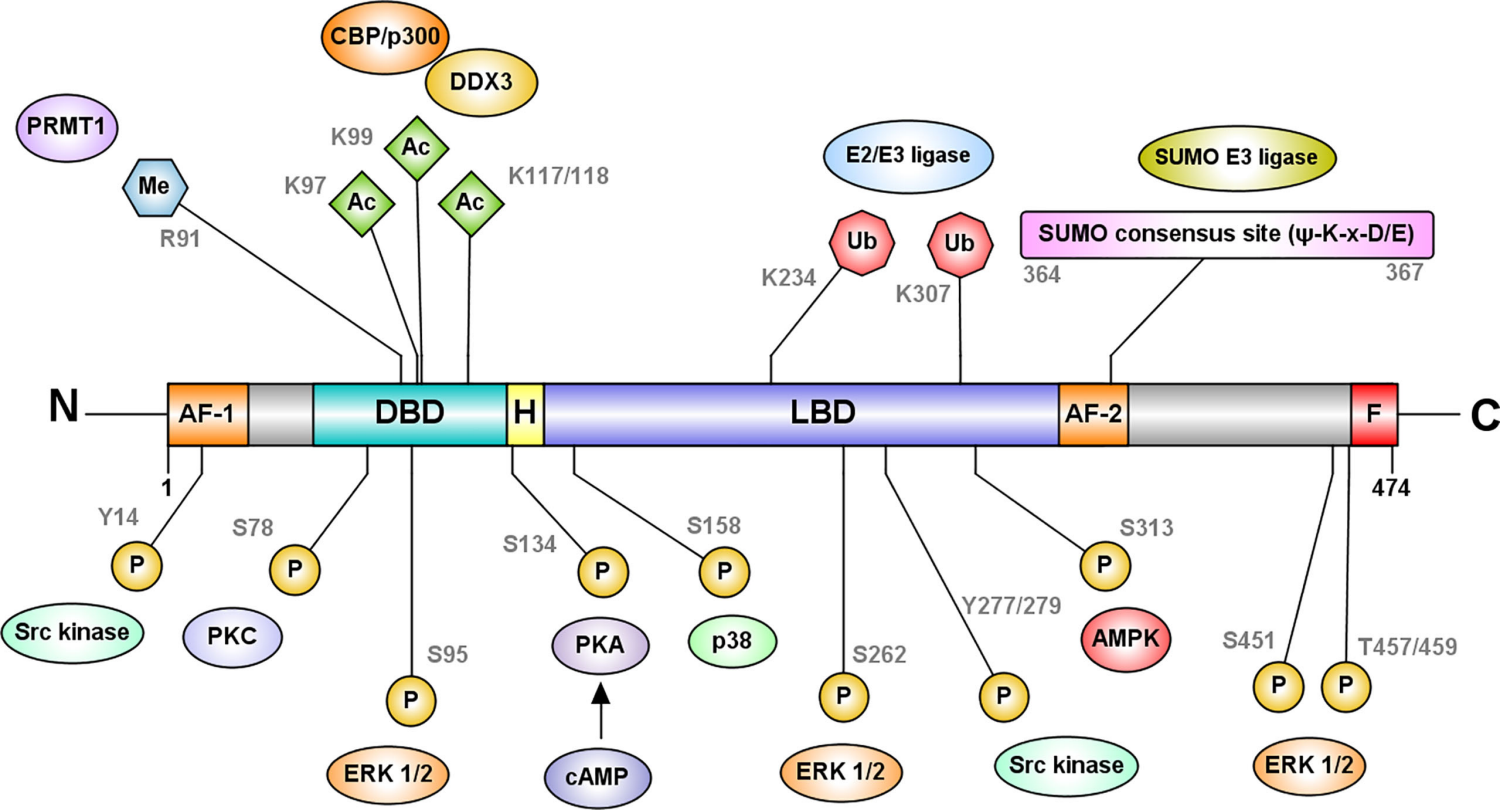
HNF4α is regulated at the protein level by post-translational modifications. The most frequently recurring protein modification is phosphorylation of serine and threonine residues by several enzymes including protein kinase C, AMP-activated kinase, ERK1/2 kinase, protein kinase A, and p38 kinase (41–45). Phosphorylation at the DBD residue S78, by protein kinase C impairs the DNA binding capacity of HNF4α and its nuclear localization (44). cAMP-induced protein kinase A phosphorylates S134 in the DBD and inhibits HNF4α’s recruitment to target genes (41). AMP activated kinase phosphorylates HNFα at S313 in the LBD and destabilizes the protein (42). ERK1/2 kinase can phosphorylate multiple residues such as S95, S262/S265, S451, and T457/T459, which reduce the transactivation capacity of HNF4α (45). Src kinase phosphorylates Y14 followed by Y277/279 of P1-HNF4α causing mislocalization of the protein to the cytoplasm (46). In contrast, p38 kinase mediated phosphorylation at S158 increases the transactivation potential of HNF4α (43). Further modifications include acetylation at DBD residues K97, K99, K117 and K118 mediated by the CREB-binding protein (CBP), in association with co-activators, p300 and DDX3 and methylation of R91 in the DBD by protein arginine methyltransferase 1 (PRMT1). Acetylation is likely required for the nuclear retention of the protein (47). HNF4α also undergoes SUMOylation at the C-terminal consensus site (Ψ-K-x-D/E) which destabilizes HNF4α in a ubiquitin-dependent manner (48). Ubiquitylation of HNF4α occurs at residues K234 and K307, and targets HNF4 for proteasomal degradation (49). Figure generated using Illustrator for Biological Sequences, Version 1.0 (33).

Post-translational modifications increase the diversity and modify the regulatory capabilities of HNF4 proteins (**Figure 4**). Several prominent post-translational modifications HNF4 undergoes are:

(i) Phosphorylation: Phosphorylation of serine and threonine residues by several enzymes including protein kinase C, AMP-activated kinase, ERK1/2 kinase, protein kinase A, Src, and p38 kinase can modify HNF4 function (41–46). Much like other nuclear receptors, HNF4 is phosphorylated in the AF-1 domain, the LBD or the DBD. Based on the domain where this modification occurs, the functional impact on HNF4 activity can vary between changes in dimerization, transactivation, DNA binding and cofactor recruitment. As the nuclear localization signals and export signals are in these domains, HNF4’s transport into the nucleus could also be affected by phosphorylation. For instance, phosphorylation of P1-HNF4α by Src kinase causes mislocalization of the protein to the cytoplasm and complete loss of protein stability (46).
(ii) Acetylation: Acetylation at DBD residues K97, K99, K117 and K118 is mediated by the CREB-binding protein (CBP), in association with co-activators, p300 and DDX3, and increases the DNA binding capacity of HNF4α. The region of acetylation overlaps with the NLS, suggesting that acetylation is required for the nuclear retention of the protein (47).
(iii) Methylation: Methylation of R91 in the DBD by protein arginine methyltransferase 1 (PRMT1) also increases the transcriptional activity of HNF4α. PRMT1 also functions as a HNF4α co-activator and functions synergistically with other co-activators at HNF4α target promoters (50).
(iv) SUMOylation: In addition, HNF4α also undergoes ubiquitination and SUMOylation. Successive SUMOylations at the C-terminal residues K365 and D367 destabilize HNF4α in a ubiquitin-dependent manner (48).
(v) Ubiquitylation: Ubiquitylation occurs at residues K234 and K307, and targets HNF4 for proteasomal degradation (49).

Considering that post-translational modifications in other proteins are generally isoform-specific, we can assume that the same paradigm might apply to HNF4. Understanding this can be a focal point for future investigations.

### An intriguing form of regulation: intestinal microbiota suppress HNF4α

The intestinal microbiota exerts a fascinating form of regulation by suppressing the expression and activity of Hnf4α (hepatocyte nuclear factor 4 alpha) in the intestines of various organisms, including zebrafish and mice.

In zebrafish, studies have shown that following microbial colonization, the transcriptional expression of hundreds of genes is suppressed, with nearly half of them being dependent on Hnf4α (51, 52). Interestingly, reduced Hnf4α binding, as measured by ChIP, is a key feature of microbial-suppressed genes, suggesting that Hnf4α activity itself is suppressed by the microbiome. Researchers have found that Hnf4α can mediate the reactivation of microbiota-suppressed genes by specifically binding to and activating transcriptional enhancers, such as the in3.4 enhancer located at the angptl4 gene in zebrafish, as demonstrated through GFP reporter assays. Similar effects have been observed in genomic analyses of gnotobiotic mice, where microbial colonization leads to broad changes in enhancer activation and a reduction in the occupancy of HNF4α and HNF4γ on their respective target genes. The interaction between HNF44α and other putative transcription factors, including GATA, PDX1, and HOXC9, may play a role in mediating the microbial control of intestinal gene expression (51). The downstream effects of the alliance between HNF4α and the microbiota have been explored in mouse jejunal epithelial cells. Through multi-omics analyses, researchers have illustrated that suppression of *Hnf4a* by a combination of the microbiota and a high-fat meal promotes a proliferative phenotype, leading to a loss of homeostatic balance (53). Moreover, interspecies meta-analysis connected these findings to humans with the discovery that HNF4α-activated microbiota-suppressed gene sets were associated with obesity related traits and inflammatory bowel diseases (51). Clearly there exists a conserved regulatory relationship between HNF4α, its transcription factor network and microbiota in maintaining intestinal homeostasis. The microbiota-HNF4α relationship may have important implications for understanding and addressing human intestinal diseases.

## Unraveling the role of HNF4 in the intestine: key perspectives and findings

HNF4 is of paramount importance in the regulation of multiple facets of intestinal development, function, and homeostasis. In the following section, we explore and elucidate some of the major roles it plays in these critical processes.

### HNF4 plays an essential role during intestinal development

**Figure 5:**
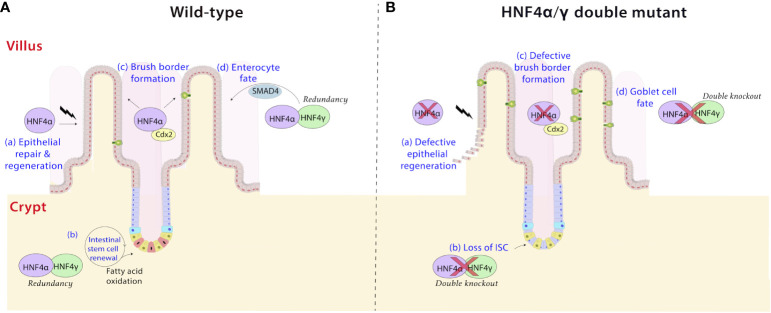
HNF4 plays diverse roles in the intestine. (a) HNF4α is a driver of repair and regeneration mechanisms in the intestine (58). It controls crypt survival and proliferating cell survival, which would allow epithelial repopulation **(A)**, a phenomenon which is lost in HNF4α mutants **(B)**. (b) HNF4α and HNF4γ are highly expressed in Lgr5+ intestinal stem cells (ISCs) and are required for ISC maintenance and renewal **(A)** (59). Dysregulation of fatty acid oxidation in stem cells causes exhaustion, loss of ISC and premature lineage commitment (Panel B) (60, 61). This effect is pronounced in HNF4α/γ double mutants, implying redundancy masks the phenotype in single mutants. (c) The HNF4α-CDX2 pair controls genes responsible for formation of the apical brush border in the intestine (62). HNF4 functions as a conserved and universal regulator of brush border genes by likely operating as a mechanosignaling sensor detecting changes in actin fibers and brush border transcripts upon mechanical stress (63). Electron microscopy cross sections through microvilli reveal a larger brush border diameter and shorter height in HNF4α/γ double mutant mice compared to wild type **(A, B)**. (d) HNF4 paralogs, in association with the BMP/SMAD pathway mediate the formation of enterocytes in the villi. HNF4 and SMAD4 reciprocally activate each other’s expression in villi *in vivo* and in organoid models *ex vivo*, and disruption of this feed-forward loop results in loss of enterocyte fate in favor of a goblet cell fate **(B)** (17).

E8.5 is the inception point for mouse intestinal development. At this stage, the visceral endoderm invaginates to form a common gut tube that includes the foregut and the hindgut. Expression of CDX2 is subsequently required to specify the intestine from the more antral GI tissues (54–56). Until E14.5, the developing intestine is comprised of rapidly proliferating, pseudostratified epithelium. Between E14.5 and E16.5, villus morphogenesis and maturation occur. Analysis of chromatin landscapes using ATAC-seq at different time points suggest a transition in genome occupancy across this developmental transition and indicates that HNF4 binding is abundant at accessible genomic regions upon intestinal maturation. Facilitated by the *Shh-Cre* driver, inactivation of both HNF4 paralogs in the embryonic endoderm demonstrates that HNF4 factors are largely dispensable in the developing intestine until villus elongation and maturation at E16.5 (57). It should be noted that the intestine remains properly specified in the *Shh-Cre, Hnf4* double mutants, indicating that HNF4 factors function only in the maturation of the tissue, rather than its developmental specification, a role unmistakably attributed to the transcription factor, CDX2 (54, 57). CDX2 functions upstream of HNF4, with decreased *Hnf4* expression in the *Cdx2* knockout intestine. The HNF4α-CDX2 pair occupy shared genomic regulatory sites to promote chromatin accessibility and gene expression in the maturing intestine. Together they control genes responsible for formation of the apical brush border (**Figure 5**) and absorption of dietary lipids in the intestine (62). Further studies in human cell lines *in vitro* reveal the HNF4 regulatory cascade is balanced by either COUP-TFII or HNF3α, both of which can repress gene expression of *HNF4A* (64, 65). Retinoic acid receptors RXR-RAR compete for the same binding site as COUP-TFII and can alleviate the repressive effects (65).

During later stages of development, several signaling cascades which control patterning of the gut have been shown to be influenced by HNF4 (66, 67). For instance, in crypts, which develop postnatally in mice, HNF4α sequesters TCF4, an effector of Wnt/*β*-catenin signaling, whose inactivation is required to control excessive proliferation (16, 68). Similarly, the activation of the Notch pathway induces the transcription factors HES1 and MATH1, to commit epithelial cells to the absorptive or secretory lineages, respectively. Ablation of HNF4 skews the phenotype towards secretory cells by increasing MATH1 levels (16). Further, during colon development, use of the *Foxa3-Cre* driver, which is active before the onset of *Shh-Cre*, found that HNFα is required for the formation of colonic crypts. This phenotype was not observed in the *Shh-Cre* model (57), which could either reflect differences in the onset of Cre activity between the two models, or secondary consequences of loss of HNF4 in the liver of the *Foxa3-Cre* model (69, 70). Differences in HNF4α sequence determinants, binding motifs and absolute dosage among different species may, in some measure, account for inter-species developmental variations among tissues of the gastrointestinal system (71, 72).

### HNF4 is a key regulator of intestinal metabolism

The first and foremost role of the intestine is digestion and absorption of nutrients, primarily dietary lipids. This process primarily occurs in enterocytes of the villi (73). HNF4α controls fatty acid uptake by enterocytes, cellular lipid transport, and apolipoprotein synthesis (74, 75). In Drosophila, dHNF4 functions as a sensor for free fatty acids, and is essential for fatty acid oxidation (FAO). dHNF4 null mutant larvae retain increased levels of long-chain fatty acids in their midgut and fat body, suggesting an inability to mobilize stored fat for energy (76). In higher mammals, HNF4 binds intermediate metabolites of lipid metabolic pathways. Genes associated with acyl coenzyme A metabolism were also identified as putative HNF4α targets (21, 77). In fact, the loss of HNF4 factors in mice leads to decreased transcript levels of genes involved in almost every step of FAO (59). This activity of HNF4 factors in regulating FAO is important for supporting intestinal stem cell renewal (further explored below).

HNF4α also affects incretin metabolism. Incretins are two hormones produced by the enteroendocrine cells of the villi, which augment insulin release by pancreatic β-cells. Incretins have broad roles in glucose metabolism, cardiovascular function, bone metabolism, and triglyceride storage in adipose tissue (78). The precise impact of HNF4α on one of the incretins, glucagon-like peptide-1 (GLP-1), is not yet well understood. However, studies in intestinal *Hnf4a*-deficient mice have revealed that HNF4α regulates the production of the other incretin, glucose-dependent insulinotropic polypeptide (GIP) in association with GATA-4 (79). Interestingly, the deletion of intestinal *Hnf4a* does not directly affect enterocyte lipid metabolism but has a broader whole-body effect, resulting in a resistance to diet-induced obesity (DIO) (80). Intestinal *Hnf4a-*mutant mice showed a preference for utilizing fat as an energy substrate and experienced significant changes in energy metabolism within white adipose tissue (WAT). Notably, the WAT underwent beiging, a process where it acquired characteristics similar to brown adipose tissue. When a stabilized analog of GIP was introduced, it was able to rescue the DIO resistance phenotype. This suggests that the impairment in fat-induced GIP release is a key mechanism contributing to the DIO resistance phenotype associated with intestinal HNF4α deletion. The reintroduction of the GIP analog restores the proper signaling and metabolic response, resulting in the normalization of the mice’s response to a high-fat diet (80).

### HNF4 and SMAD4 stabilize enterocyte identity

In the small intestine, enterocytes in the villi predominantly express P1-HNF4α while both P1 and P2 related isoforms are seen in the crypts (**Figure 2A**). Differential expression can be a contributing factor to architectural and functional variations between the two compartments. In villi, HNF4 paralogs, in association with the BMP/SMAD pathway mediate the differentiation of enterocytes from crypt progenitor cells (**Figure 5**). Transcriptome analyses between *Villin-Cre^ERT2^; Hnf4a^f/f^; Hnf4g^-/-^* compound mutant mice and *Villin-Cre^ERT2^; SMAD4^f/f^* mutant mice reveals 541 downregulated genes in common upon both SMAD4 and HNF4 inactivation. These genes are strongly associated with enterocyte functions. HNF4 and SMAD4 reciprocally activate each other’s expression in villi *in vivo* and in organoid models *ex vivo* and disruption of this feed-forward loop results in loss of enterocyte fate in favor of a goblet cell fate (17). Additional evidence that HNF4α precludes a secretory state is the decrease in functional enteroendocrine cells in intestinal *Hnf4a*-deficient mice. In this mouse model, the transcription factor NGN3 drives production of enteroendocrine cells but the lack of HNF4α prevents their full terminal differentiation (16, 81). Further, HNF4α influences the distribution of enteroendocrine cells along the crypt-villus axis driving them more towards villi than crypts (16).

### HNF4 governs key aspects of intestinal architecture while ensuring intestinal barrier integrity

The cellular organization and architecture of the intestinal epithelium is also dependent on HNF4α. HNF4α depletion causes a wider spread of intercellular tight junctions, as a result of a decline in tight junction proteins, ZO-1 and claudins 4 and 7 (16). This likely explains why mucosal erosion, loss of paracellular permeability, and susceptibility to IBD are so high in HNF4α dysfunction disorders. Likewise, HNF4α is required for the translocation of E-cadherin, an epithelial adhesion molecule, to membrane surfaces (16). Early loss of E-cadherin in enterocytes triggers anoikis in the epithelia (9, 10). Interestingly, ectopic induction of *Hnf4a* in F9 murine embryonic cells has been shown to drive the expression of tight junction proteins, occludin and claudin-7. This consequently promotes the *de novo* formation of functional tight junctions, thus maintaining epithelial integrity (82). This highlights the role of HNF4α in the regulation of tight junction proteins and its ability to preserve the barrier function of epithelial cells. It’s likely that this association with barrier integrity is a major reason why mutations in the human *HNF4A* locus are associated with heritable risk for ulcerative colitis (83–85).

Additionally, HNF4 controls an extensive battery of genes associated with the brush border (**Figure 5**). Recent studies show expression of brush border markers, such as alkaline phosphatase and *Espn*, are downregulated upon loss of both intestinal *Hnf4* factors in mice. Electron microscopy cross sections through microvilli reveal a larger diameter and shorter height in *Hnf4a/g* double mutant mice compared to the organized, uniform microvillar arrays in the wild type. Mouse genetic experiments also show that HNF4α controls expression of brush border genes in other tissues *in vivo*, including the proximal tubules of the kidney and in the villous structures protruding from the proximal murine yolk sac (63). Clearly, HNF4 functions as a conserved and universal regulator of brush border genes. Brush border anomalies are reported in many human intestinal disorders, including Crohn’s disease, celiac disease, and congenital sodium diarrhea (86–88).

### Epithelium renewal and repair in the intestine is dependent on HNF4

The renewal of the intestinal epithelium is accomplished by the Lgr5+ stem cell population located in the crypts. These cells divide every 24 hours, generating rapidly proliferative progenitors, which differentiate into various cell types of the intestine, a process which requires high energy expenditure. *Hnf4a* and *Hnf4g* are highly expressed in intestinal stem cells (ISC) and have been determined to be necessary for ISC maintenance and renewal (59). HNF4α and HNF4γ bind to endogenous fatty acids (36, 37), such as linoleic acid in the case of HNF4α (39), and activate genes corresponding to the fatty acid oxidation (FAO) pathway; a process essential for ISC renewal (89, 90). ChIP-seq experiments conducted in wild-type mice have provided evidence that HNF4 directly binds to genes involved in FAO, including *Abcd1*, *Acox1*, and *Ehhadh* (17, 59). Further, RNA-seq experiments in *Hnf4a/g* double mutant mice revealed a significant decrease in the expression of FAO genes, indicating that HNF4 is indeed involved in activating their expression (17, 59). Loss of HNF4 paralogs impairs ISC renewal, instead pushing ISCs towards lineage commitment leading to an increased number of transit-amplifying cells and an escalation of cell proliferation. In intestinal organoid assays, metabolic intervention with acetate or dichloroacetate has been shown to have a beneficial effect on ISC loss in *Hnf4a/g* double mutants. By providing these exogenous 2-carbon fatty acids, the metabolic intervention helps to compensate for the deficiency in FAO observed in the *Hnf4a/g* double mutants. The homeostasis of ISCs and other concomitant intestinal cell types, is therefore heavily dependent on HNF4 and its regulation of metabolic pathways (59).

The intestinal epithelium exhibits remarkable plasticity, with the ability to regenerate within 72 hours following damage (58). HNF4α is a likely driver of these repair mechanisms in intestinal organoids and mutant mouse models (58) (**Figure 5**). Studies *ex vivo* in organoids show HNF4α helps establish organoids from clusters of broken up epithelial cells during passaging. Further, *in vivo*, HNF4 was shown to control epithelial repopulation after irradiation in mice. Intestinal damage inflicted by sublethal radiation leads to a reduction in the rate of amino acid and lipid metabolism, both of which are processes dependent on HNF4 in the intestine. There could be connections in the intestine between activation of p53 and repression of HNF4 activity to support this change in damaged intestine, as this connection has been documented in liver cells (91, 92). As expected, intestinal *Hnf4a* mutant mice had fewer proliferating cells per surviving crypt. This and previous studies of HNF4 on stem cell renewal illustrate the critical role HNF4α orchestrates in crypt and proliferating cell survival (59, 76).

### Paneth cell homeostasis is dependent on HNF4

HNF4α plays a pivotal role in maintaining Paneth cell homeostasis and epithelial renewal in the intestine. Recent studies have provided further insights into the dependence of these processes on HNF4α and its relationship with Wnt signaling.

Studies using murine jejunal enteroids lacking *Hnf4a* have shown that the addition of exogenous Wnt3a or co-culture with mesenchymal cells can rescue the phenotype (93). Notably, immunofluorescence assays for lysozyme showed a reduction of Paneth cells in the *Hnf4a-*deficient enteroids co-cultured with mesenchyme but a restoration of the Paneth cell signature in those rescued with Wnt3a. The likely reason for this is the dependence of Paneth cells on Wnt signaling (4). Transcriptomic analyses of *Hnf4a*-deficient enteroids have revealed that both Wnt3a supplementation and mesenchymal cell co-culture can rescue a significant proportion of the transcriptomic changes observed in the absence of HNF4α - Wnt3a rescue accounted for approximately 89% of the changes, while mesenchymal cell co-culture rescued around 91%. Further, in studies using engineered intestinal epithelial cell lines expressing HNF4α2, researchers have demonstrated the direct binding of HNF4α2 to the *Wnt3* gene. Overall, these findings highlight the intricate relationship between HNF4α, Wnt signaling, and Paneth cell development. HNF4α acts as an upstream transcriptional regulator of the *Wnt3* gene, affecting autocrine epithelial Wnt3 signaling and contributing to Paneth cell homeostasis in the intestine (7, 93).

## HNF4α and HNF4γ exhibit genetic redundancy in the intestine

While HNF4α is a key factor in maintaining intestinal homeostasis, its study is incomplete without decoding the role of its intestine-restricted paralog HNF4γ (**Figure 1**). HNF4γ functionally overlaps with HNF4α and compensates for its loss, pointing to genetic redundancy between the two factors. In mice, HNF4α and HNF4γ show similar DNA binding profiles, along with overlapping *in situ* expression patterns in the intestine. Ablation of either *Hnf4a* or *Hnf4g* alone yield fertile, developmentally normal offspring, indicating each individual paralog is largely dispensable. However, a tamoxifen-inducible double knockout of *Hnf4a* and *Hnf4g* results in striking changes in intestinal structure and function (17). Apart from demonstrating an emaciated phenotype, the *Hnf4a/g* double mutant mice develop fluid-filled intestines and die within 4-5 days of phenotype onset, suggesting redundant functions between the HNF4 paralogs (17).

Loss of either HNF4α (16, 94) or HNF4γ (95, 96) paralogs in mice show relatively modest phenotypic changes due to compensation by the other, thus buffering against fluctuations in overall *Hnf4* gene expression. Redundancy would allow one paralog to accumulate mutations if the other is functionally intact. Loss of both paralogs, however, would cause detrimental changes in intestinal structure and function with loss in homeostatic balance (17). In this section, we delve into the functionalities and impacts of HNF4 redundancy within the intestinal system.

### Redundancy during zebrafish gut development

In zebrafish larvae, *hnf4a* and *hnf4g* show partial genetic redundancy. Germline loss of *hnf4a* alone has a stronger impact on intestinal processes than loss of *hnf4g* and these changes are further exacerbated in a *hnf4a/hnf4g* double mutant model. The double mutant ultimately results in larval lethality between 6 to 14 days post fertilization (97). Moreover, zebrafish and other lower vertebrates like *Xenopus laevis* express a third paralog, *hnf4b* (98, 99). *hnf4a* and *hnf4b* are reported to redundantly regulate genes involved in yolk lipid mobilization to the embryonic body during early zebrafish development; a function which might be conserved in other egg-laying vertebrates but lost in higher vertebrates due to the lack of *hnf4b*. Double mutants of *hnf4a* and *hnf4b* or triple mutants of *hnf4a*, *hnf4g* and *hnf4b* in zebrafish are not viable, further pointing to multiple layers of redundancy between paralogs (97).

### Redundancy during murine intestinal development

HNF4 paralogs are also redundant in the developing gut. HNF4 DNA-binding motifs are more prevalent at accessible chromatin regions at E14.5-E18.5, which corresponds to the period of villus morphogenesis, compared to earlier stages of intestinal development. Ablating *Hnf4a* in a *Hnf4g^-/-^* background using an *Shh-Cre* driver leads to shorter villi, indicating HNF4 is redundantly required for villus elongation and extension into the gut lumen. As mentioned previously, brush border formation is also affected in the double mutant (57). Loss of HNF4α alone in the developing intestinal epithelium does not result in the same pronounced morphological effects (69, 94).

### Redundancy is required to maintain intestinal homeostasis

The redundant functions of HNF4 paralogs have also been elucidated in the context of intestinal homeostasis. As previously discussed, HNF4α and HNF4γ are necessary for ISC maintenance and renewal (59) (**Figure 5**). Single knockouts of either *Hnf4a* or *Hnf4g* show only minor phenotypic changes with no effect on ISC maintenance. Conversely, *Hnf4a/g* double mutants show compromised β-oxidation and increased apoptosis of ISCs in the crypt base, causing disruption in their renewal (59). It is noteworthy that the redundancy of HNF4 paralogs clearly extends beyond the fetal stage and coordinates metabolic regulation in the mature intestine.

### Redundancy during intestinal cellular differentiation programs

Furthermore, HNF4 paralogs redundantly regulate cellular identity in the intestine. HNF4α acts redundantly with HNF4γ, to activate and maintain distal enhancer chromatin and upregulate programs of cellular differentiation. As the most abundant intestinal cell type, enterocytes are a key beneficiary of such mechanisms. Indeed, as mentioned previously, a positive regulatory circuit between HNF4 and the BMP/SMAD signaling pathway promotes enterocyte identity. Disruption of this module requires ablation of *Smad4* or both *Hnf4* paralogs for the increased goblet cell phenotype to be established (17) (**Figure 5**). Cellular proliferation and differentiation are thus tightly regulated in the intestinal epithelium via mechanisms of feedback and functional redundancy.

### Redundancy during intestinal immune signaling

Recent studies in mice have highlighted the crucial role of HNF4α in mediating communication between intraepithelial lymphocytes (IELs) and epithelial cells (100). This communication relies on the HNF4α-dependent regulation of immune signaling molecules, including Btnl1, Btnl6, H2-T3, and Clec2e. In the colon and distal small intestine, the expression of *Btnl1* and *Btnl6* is controlled by HNF4α. However, in the duodenum and jejunum, HNF4γ takes over the regulatory role. ChIP-qPCR analyses have revealed that HNF4γ strongly binds to the promoters of *Btnl1* and *Btnl6*, even in the absence of HNF4α. Furthermore, in duodenal epithelial cells lacking both *Hnf4a* and *Hnf4g*, there is a significant reduction in the levels of Btnl1 and Btnl6 compared to single mutant models of *Hnf4a* or *Hnf4g* (17, 100). This suggests that HNF4γ acts redundantly with HNF4α, serving as a substitute in the regulation of genes involved in the communication between IELs and intestinal epithelial cells.

### HNF4 redundancy may have a regulatory role in modulating circadian behaviors

Of note, HNF4α and HNF4γ contribute to the maintenance of tissue-specific circadian oscillations in both intestinal and liver cells, by trans-repressing the activity of the master circadian transcription factor CLOCK:BMAL1 (101). This suggests that HNF4 transcription factors may be redundantly involved in shaping the circadian clock’s molecular mechanisms. However, whether this non-canonical activity is redundant or not is debatable. Nevertheless, the redundancy between HNF4α and HNF4γ may provide a selective advantage for HNF4 in the control of intestinal gene expression.

## HNF4 variants are associated with disease phenotypes in humans

**Figure 6:**
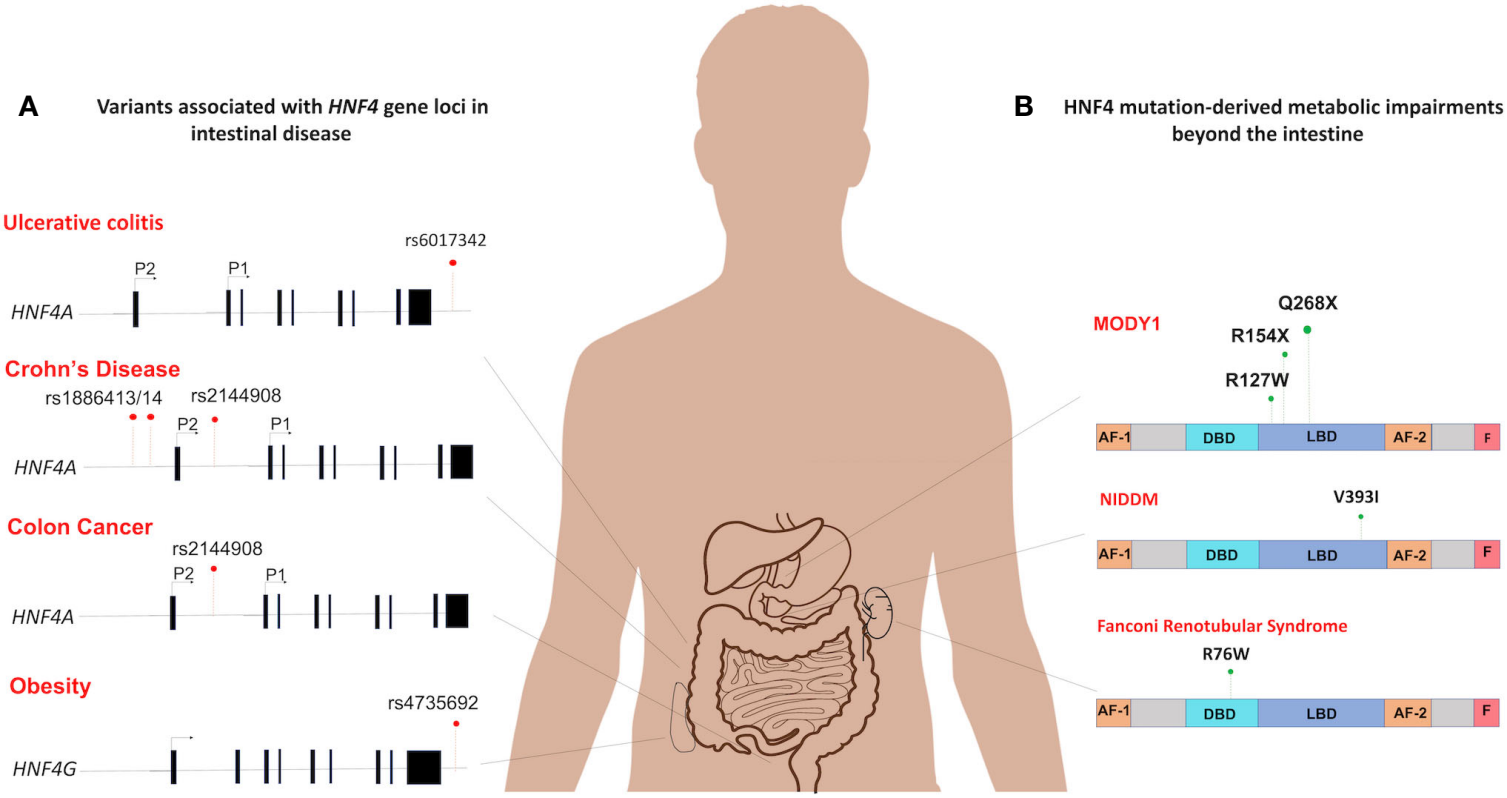
Spectrum of HNF4 dysfunctions in the intestine and beyond. **(A)** The mutational distribution of *HNF4* generates 4 main disease phenotypes. An SNP in in the 3’-UTR of *HNF4A* has been associated with ulcerative colitis (rs6017342) (83–85). Three SNPs each are significantly associated with increased susceptibility to childhood-onset Crohn’s disease (102) (rs2144908, rs1884613 and rs1884614) and colon cancer (46) (rs6031602, rs1063239, and rs6093980). A single SNP in the human locus of *HNF4G* (rs4735692) has been associated with obesity (103). **(B)** At the protein level, HNF4α dysfunctions are seen as metabolic impairments such as MODY1 (104), and Non-insulin dependent diabetes mellitus (NIDDM) (105). In MODY1, a low frequency missense mutation in HNF4α, Q268X, results in the deletion of 187 C-terminal amino acids (106), whereas a V393I substitution causes increase in susceptibility to NIDDM (107). Also, Fanconi renotubular syndrome is a unique phenotype comprising both MODY1 and atypical Fanconi syndrome, which occurs due to a heterozygous missense mutation, R76W in HNF4α (108).

Mutations in HNF4 are a pathophysiological feature of many human diseases (**Figure 5**). While the mutational spectrum tends to be variable, most reported cases of disease-associated mutations are often deleterious and involve loss of function. Understanding HNF4 disease phenotypes is further complicated by the functional redundancy between HNF4α and HNFγ, as phenotypes might only manifest under a specified set of circumstances. Nevertheless, a comprehensive review of clinical phenotypes attributed to HNF4 mutations reveals common themes of loss of function and haploinsufficiency. Some of the most well-known manifestations of HNF4α mutations are seen as perturbations of glucose metabolism, such as in Type 1 maturity onset diabetes of the young (MODY1) and non-insulin dependent diabetes mellitus (NIDDM) (**Figure 6**). MODY is an adolescent-onset, monogenic and hereditary form of Type 1 diabetes (104), whereas NIDDM is adult-onset, multifactorial and occurs due to an imbalance between insulin sensitivity and secretion (105). In MODY1, a low frequency missense mutation in HNF4α, Q268X, results in the deletion of 187 C-terminal amino acids, leading to mislocalization of the protein, loss of transactivation activity and failure to dimerize and bind DNA (109, 110). In NIDDM, a V393I substitution in HNF4α results in a decrease in transcriptional activity and insulin secretion (107). Additionally, Fanconi renotubular syndrome is a unique phenotype comprising both MODY1 and atypical Fanconi syndrome, which occurs due to a heterozygous missense mutation, R76W in HNF4α. Such patients present with fetal macrosomia and neonatal hypoglycemia associated with hyperinsulinemia (108). The differential gene expression patterns of HNF4 across various organs also helps account for the wide range of disease manifestations.

HNF4-related diseases specifically affecting the intestine are primarily associated with mutations or variations in the *HNF4* gene, leading to impaired regulation of various aspects of intestinal development, function, and homeostasis. The specific manifestations of HNF4-related intestinal diseases can vary and may include disruptions in metabolism, architecture, epithelial cell differentiation, and other essential processes within the intestine. In this section, we explore different diseases and their connection to HNF4 in the context of intestinal health.

### HNF4 is required to avert a chronic inflammatory state in the intestine

In the intestine, dysregulation of HNF4α underlies a multitude of disease phenotypes, notably, inflammatory bowel disease (IBD). Indeed, *Hnf4a* is considered an IBD susceptibility gene (83, 111, 112). Crohn’s disease and ulcerative colitis are the most common forms of IBD and HNF4α has been implicated in both. Previous studies using a DSS-induced colitis mouse model have provided evidence that conditional deletion of *Hnf4a* leads to a decrease in body mass and an increase in intestinal permeability (110). However, another study has shown that HNF4α deficiency did not result in clinical disease or dysbiosis in young mice. Instead, it caused an early disruption of intestinal homeostasis, which subsequently increased the susceptibility to colitis in older animals (100). HNF4α, is therefore necessary to maintain the integrity of the mucosal epithelial barrier. Separate studies in exon swap mice genetically engineered to express only *P1-Hnf4a* or *P2-Hnf4a* show that intestinal barrier functions mediated by HNF4α are isoform-specific. Mice producing P1-HNF4α proteins were less susceptible to colitis whereas mice ectopically expressing only P2-HNF4α were more inclined to develop colitis. The *P2-Hnf4α* mice had higher levels of RELMβ, a cytokine which activates innate immune responses upon disruption of intestinal barrier function (29). Such mice also exhibit imbalances in electrogenic sodium and chloride secretion and impaired water absorption in the colonic mucosa, two of the main triggers of the diarrheal symptoms of colitis (29, 113). Results implicating HNF4α in IBD are bolstered by evidence from genome-wide association studies in geographically diverse cohorts of ulcerative colitis patients, which identify *HNF4A* as a major susceptibility locus. A single nucleotide polymorphism (SNP), rs6017342, in the 3’-UTR of *HNF4A* causes increased susceptibility to ulcerative colitis (83–85) (**Figure 6**). Furthermore, three additional SNPs (rs2144908, rs1884613 and rs1884614) are significantly associated with increased susceptibility to childhood-onset Crohn’s disease (102) (**Figure 6**). In addition, human intestinal biopsies from patients with IBD show decreased expression of HNF4α (111). Recent work demonstrating a potential treatment strategy against IBD uses HNF4α to induce NHE3 (Na^+^/H^+^ exchanger isoform 3), which can restore Na^+^ absorption in the intestine (114). HNF4α integrity is thus crucial and plays a protective role against predisposition to IBD following chronic intestinal inflammation.

### HNF4 alterations can increase the risk of colon carcinomas

A diagnosis of IBD is a significant risk factor for developing colorectal cancer (CRC) and colitis-associated cancer (CAC), predominantly due to the pro-neoplastic effects of chronic inflammatory insults. Thus, by default, the protective effect of HNFα in IBD can be extended to intestinal cancers as well. In a mouse model of CAC, expression of only P1-HNF4α is associated with lower tumor burden, while the opposite effect is seen during ectopic expression of only P2-HNF4α. The tumor-inducing effect of P2-HNF4α is attributed to the absence of P1-HNF4α in these mice, thus, suggesting a protective role for P1-HNF4α against cancer (29). A more direct relationship between HNF4α and CRC in humans can be seen by three functional variants of *HNF4A* (rs6031602, rs1063239, and rs6093980), the expression of which could increase an individual’s susceptibility to Src-kinase mediated CRC (46) (**Figure 6**). Investigation into the molecular connections between HNF4 and Src-kinase mediated CRC reveals that HNF4α again mediates susceptibility in an isoform dependent. Phosphorylation of three residues in P1-HNF4α by Src kinase leads to protein instability and transcriptional dysregulation. This action by Src is specific to P1-HNF4α as P2-HNF4α lacks a Y14 residue in the AF-1 which is the first residue phosphorylated followed by 2 residues in the LBD (46). Three SNPs in the human HNF4α protein, two of which are in the HNF4α F domain which interacts with the Src SH3 domain, increase phosphorylation by Src and decrease HNF4α protein stability and function, suggesting that individuals with those variants may be more susceptible to Src-mediated effects. Indeed, there is an 80% loss of nuclear P1-HNF4α in >450 analyzed human Stage III colon tumors which correlates with active Src (46). Further, a loss of P1- but not P2-HNF4α has been observed in many other human cancers, including renal carcinoma and hepatocellular carcinoma (28, 112, 115, 116). Rather than an active role promoting cell proliferation, P2-HNF4α is merely thought to play a permissive role, consistent with its expression in the distal proliferative compartment of the colonic crypt (29).

Vuong et al. also explored the differential effects of the HNF4α isoforms on tumor growth in human CRC (117). They used Tet-On-Inducible HCT116 colon cancer cell lines expressing either HNF4α2 or HNF4α8 isoforms that were injected in immunocompromised mice. After 8 days of development, mice were fed a diet supplemented with doxycycline to induce HNF4α expression. Tumors with HNF4α2 were significantly smaller compared to the parental controls suggesting that HNF4α2 is an active tumor suppressor in CRC. Conversely, the HNF4α8 induced tumors were larger and showed higher invasive index, making it permissive of the cancer state. Ontology analyses on genes specifically upregulated by HNF4α2 showed terms such as cell death and growth inhibition, whereas HNF4α8 upregulated genes were involved in cell proliferation. Mechanistically, HNF4α8 is believed to preferentially modulate the Wnt/*β*-catenin/TCF4 and AP-1 pathways. Taken together, these results help explain the contradictory notion that HNF4α is both a tumor suppressor and at least somewhat oncogenic; the isoforms perform distinct roles in the HCT116 cells by interacting with different co-regulators.

### HNF4 regulates intestinal entry of SARS-CoV2

Severe acute respiratory syndrome coronavirus 2 (SARS-CoV-2), the causative organism of coronavirus disease 2019 (COVID-19), has been implicated in triggering gastrointestinal symptoms by infecting the epithelial cells lining the gastrointestinal tract (118). ACE2 functions as the viral receptor in absorptive enterocytes and TMPRSS2 is involved in viral spike protein priming (119). Using epigenomic approaches Chen et al. identified HNF4 factors, in association with CDX2, SMAD4, and GATA factors, impact *Ace2* and *Tmprss2* gene expression (120). All four factors coordinately alter chromatin structure and activate intestinal *Ace2* expression, while conversely suppressing *Tmprss2*. Further, H3K4me3-targeted HiChIP assays demonstrate multiple contacts between COVID-19-related gene promoters and these regulatory elements (120). Differential usage of HNF4 and its accessory elements, along with variability in their binding sites could be one possible explanation for the wide-ranging disparity in the susceptibility to COVID-19. Better understanding of intestinal regulatory mechanisms may help in the development of therapies to reduce the systemic effects of COVID-19 and lower the severity of its associated symptoms.

### Disease phenotypes attributed to HNF4γ are ill-defined

Interestingly, very few reports exist of disease phenotypes attributed to HNF4γ alterations. A single nucleotide variant in the human locus of the *HNF4G* gene (rs4735692) has been associated with obesity (103) (**Figure 6**). Systematic association mapping of individuals with IBD reveals a mild association with a SNP of the *HNF4G* intron (121). Moreover, in a clinical study of ulcerative colitis patients, HNF4γ2 is significantly downregulated, hinting at a protective role for HNF4γ2 against ulcerative colitis (31, 122). A homozygous deletion of *HNF4G* in mice resulted in animals with a higher body weight despite having reduced intake of food and water. Additionally, they exhibited less nocturnal activity, and were less inclined to build nests, compared to their wild-type littermates (95). Also, studies have shown *HNF4G* expression increases in some bladder cancer phenotypes and in intestinal metaplasia, which is highly associated with gastric cancer (123, 124). One study has named HNF4γ as an oncoprotein in bladder and lung carcinomas (125). It remains poorly understood how the principle of redundancy between HNF4α and HNF4γ can be applied to such disease phenotypes.

## HNF4 has diagnostic and therapeutic potential in the intestine and beyond

HNF4 has diagnostic potential in the intestine as a marker for intestinal differentiation and function. Its expression and activity can serve as indicators of the intestinal cellular state and proper functionality. Moreover, the regulatory roles of HNF4 in gene expression and metabolic pathways make it an enticing target for therapeutic interventions in a wide range of disease states.

### HNF4 is a biomarker for gastrointestinal disease

Owing to its pivotal role in multiple regulatory pathways and disease states, HNF4 is an attractive target for the clinic, either as a diagnostic tool or a therapeutic strategy. A fair amount of recent work has supported the idea of HNF4 as a biomarker in gastrointestinal diseases. Studies show HNF4α expression as a potential diagnostic tool to distinguish between primary cancers and metastases (126, 127). Another study put forth P1-HNF4α as a prognostic indicator in Duke’s Stage C and D colon cancer patients (112). Additionally, genetic profiling of HNF4α can provide an understanding of whether polymorphisms in the human *HNF4* gene segregate with disease phenotypes. Certain individuals with SNP variants of *HNF4A* may be more susceptible to Src kinase-mediated colon cancer (46). Similarly, other SNP variants of *HNF4A* and *HNFG* can cause increased susceptibility to ulcerative colitis, Crohn’s disease and obesity (83–85, 102, 121). While HNF4 clearly has diagnostic potential, additional investigation is required to understand the complex role of the different HNF4 paralogs and isoforms in human disease.

### HNF4 shows promising potential for therapeutic applications

HNF4 is a nuclear receptor with a large, hydrophobic ligand-binding pocket potentially making it conducive for accepting antagonistic small molecules. However, its pleiotropic functions across diverse tissues could cause unintended side effects, so caution should be exercised when designing drugs against HNF4. In mammalian systems, initial evidence of the druggability of HNF4 was uncovered when a fatty acid ligand of HNF4α, linoleic acid, was found to be exchangeable and capable of reducing HNF4α’s transactivation capacity (39). Further studies in the T6PNE human pancreatic cell which stably expresses a HNF4α-dependent insulin promoter, shows this effect is limited to medium and long chain fatty acids (128, 129). Using the same *in vitro* system, a high-throughput screen of compounds identified two putative HNF4α antagonists, BIM5078 and BI6015, which bind in the same orientation as fatty acids. Both compounds bind to HNF4α with high affinity and altered expression of nearly 36% of HNF4 target genes. Further, both compounds are selectively cytotoxic to a panel of neoplastic cell lines but not their untransformed counterparts. At a dosage of 30mg/kg body weight, BI6015, in particular, is effective in mice, yet with sub-optimal pharmacokinetic properties (129). Extensive studies conducted on BI6015 have revealed that it mediates its inhibitory effects by modulating oncogenic Wnt signaling (130). Further studies have revealed *HNF4A* gene expression is downregulated by AMPK signaling and the AMPK agonist metformin, which also brings about changes in the Wnt signaling pathway. Thus, combining BI6015 with antagonists against the AMPK-HNF4α-Wnt signaling cascade represents another, possibly more efficacious, targetable pathway for drug development (131). This opens the possibility for a future where diseases mediated by dysregulations in HNF4α, could be remedied by intervention with HNF4α antagonists. Of course, there are potential applications for HNF4 agonists that could also be explored.

HNF4 also plays a more direct therapeutic role by being a component of cell-based therapies. Injection of encapsulated immortalized human hepatocytes overexpressing *HNF4A* promotes hepatic differentiation and improves liver function and survival in rat models with acute liver failure (132). Likewise, injection of mesenchymal stem cells expressing *HNF4A* in orthotopic human hepatomas in athymic, nude mice results in smaller tumors and decreased metastases through downregulation of Wnt signaling (133). However, none of these therapies, thus far, have been applied to the intestine.

## Concluding perspectives

HNF4 is clearly a hub of cellular function. Its significance lies in the fact that it is a highly conserved transcription factor with multifaceted roles across different organs of the gastrointestinal tract. The heterogeneity of its target genes can be accounted for by variations in chromatin looping patterns, post-translational modifications, interaction with coregulators and temporal changes in protein levels in different cell types and conditions. In the intestine, HNF4 plays pivotal roles in its maturation, regeneration, nutrient metabolism, and overall homeostasis. Clearly, the substantial contributions of HNF4 extend beyond its namesake organ. In the future, it will be important to study the transcriptional dynamics of HNF4 in organs such as the pancreas or kidney, to identify drugs that target HNF4, and to interpret the variability of clinical phenotypes arising from HNF4 mutations and SNPs.

While the body of research on HNF4 is large, it is also sometimes contradictory, revealing many unanswered questions. Future areas of investigation could include a deeper study into how promoter switching and alternative splicing are mediated. Isoform-specific effects of HNF4-dependent gene regulation, along with understanding the mechanisms of HNF4γ and how redundancy between HNF4 paralogs potentially works in organs other than the intestine are also important areas of future investigation. Use of conditional knockout rodent models has been fruitful in revealing HNF4 functions in the past, and combinations of mouse genetic tools with dietary interventions and other disease models associated with HNF4 activity should be similarly productive. These studies will be complemented by organoid technologies and improving methodologies in single-cell –omics approaches. CRISPR-based modifications of HNF4-target gene risk alleles and epithelial transplantations could be another area of therapeutic focus. A major challenge in the upcoming era of HNF4 research will be to distill wide-ranging diagnostic and therapeutic studies and translate the knowledge gleaned from them into clinical applications.

## Author contributions

KV wrote the draft and revised the review. KV, SR, FS, and MV revised and wrote the final version. All authors contributed to the article and approved the submitted version.
